# Supplementary material for: The combined impact of persistent infections and human genetic variation on C-reactive protein levels
Source: BMC Med. 2022 Nov 1;20:416. doi: 10.1186/s12916-022-02607-7 (PMC9623937; doi:10.1186/s12916-022-02607-7)
Supplement: Supplementary file 1 — Additional file 1: Fig. S1. Flowchart illustrating the inclusion/exclusion of individuals in the study. Orange boxes indicate the number of included antigens and pathogens. [file 12916_2022_2607_MOESM1_ESM.pdf]

Supplementary Material: The combined impact of persistent infections and human genetic variation on C-reactive protein levels

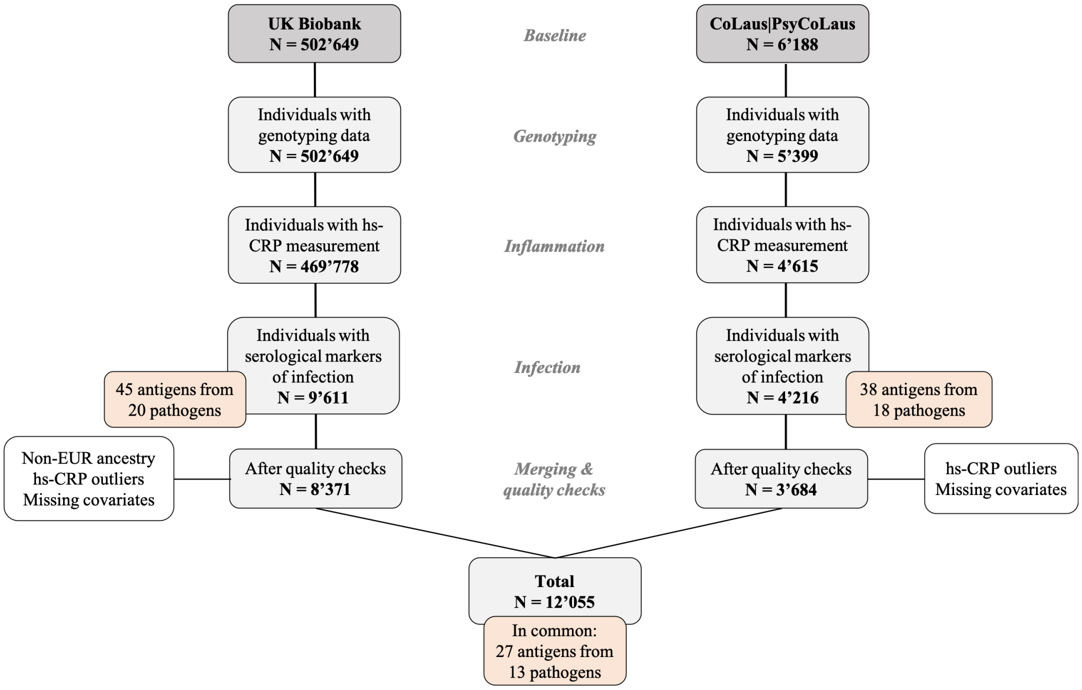

Supplementary Figure 1. Flowchart illustrating the inclusion/exclusion of individuals in the study. Orange boxes indicate the number of included antigens and pathogens.
