# Supplementary material for: The combined impact of persistent infections and human genetic variation on C-reactive protein levels
Source: BMC Med. 2022 Nov 1;20:416. doi: 10.1186/s12916-022-02607-7 (PMC9623937; doi:10.1186/s12916-022-02607-7)
Supplement: Supplementary file 2 — Additional file 2: Fig. S2. Scatterplot and regression line (with 95% confidence intervals) to describe the relationship of hs-CRP with characteristics of study participants. Relationship between hs-CRP and A) age, B) sex, C) BMI and D) polygenic risk score (PRS). For linear regressions, linear regression equation, R-squared and P-value are shown. [file 12916_2022_2607_MOESM2_ESM.pdf]

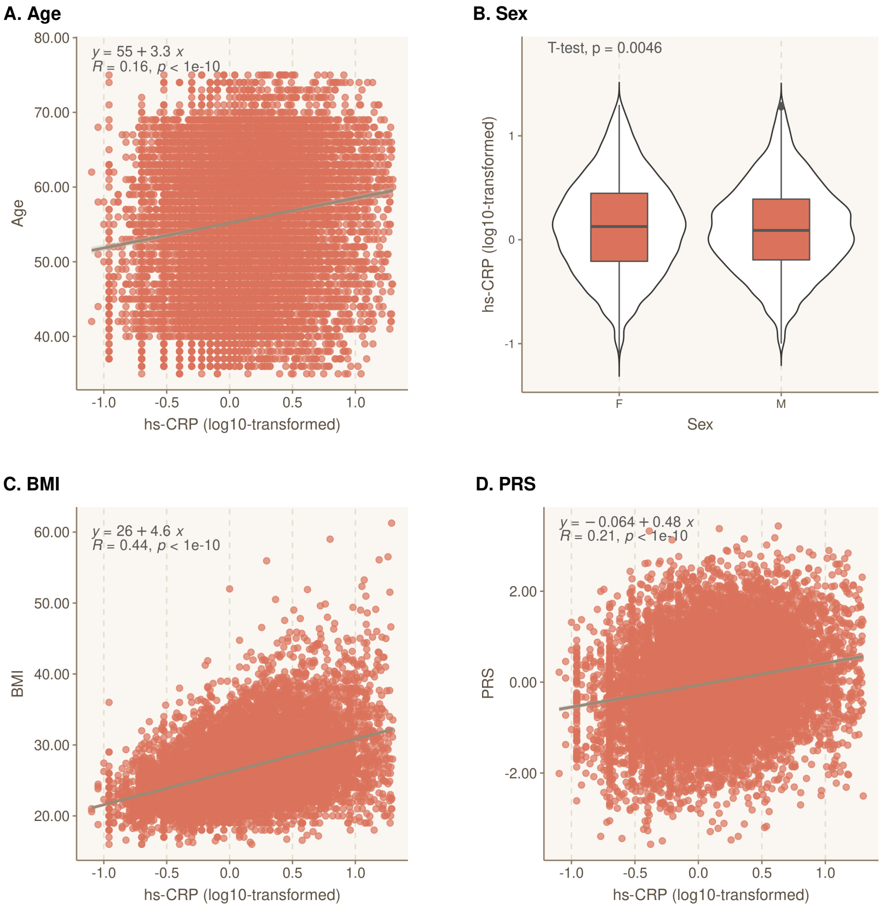

Supplementary Figure 2. Scatterplot and regression line (with 95% confidence intervals) to describe the relationship of hs-CRP with characteristics of study participants. Relationship between hs-CRP and A) age, B) sex, C) BMI and D) polygenic risk score (PRS). For linear regressions, linear regression equation, R-squared and P-value are shown.
