# Supplementary material for: The combined impact of persistent infections and human genetic variation on C-reactive protein levels
Source: BMC Med. 2022 Nov 1;20:416. doi: 10.1186/s12916-022-02607-7 (PMC9623937; doi:10.1186/s12916-022-02607-7)
Supplement: Supplementary file 3 — Additional file 3: Fig. S3. Principal component analysis (PCA) of combined genotyping data. A) PCA plot of the first ten PCs of the genotyping data. Samples are colored by cohort. B) Histogram explaining the variance of each PC component. In the histogram, the variance explained by each eigenvalue is labeled on top. [file 12916_2022_2607_MOESM3_ESM.pdf]

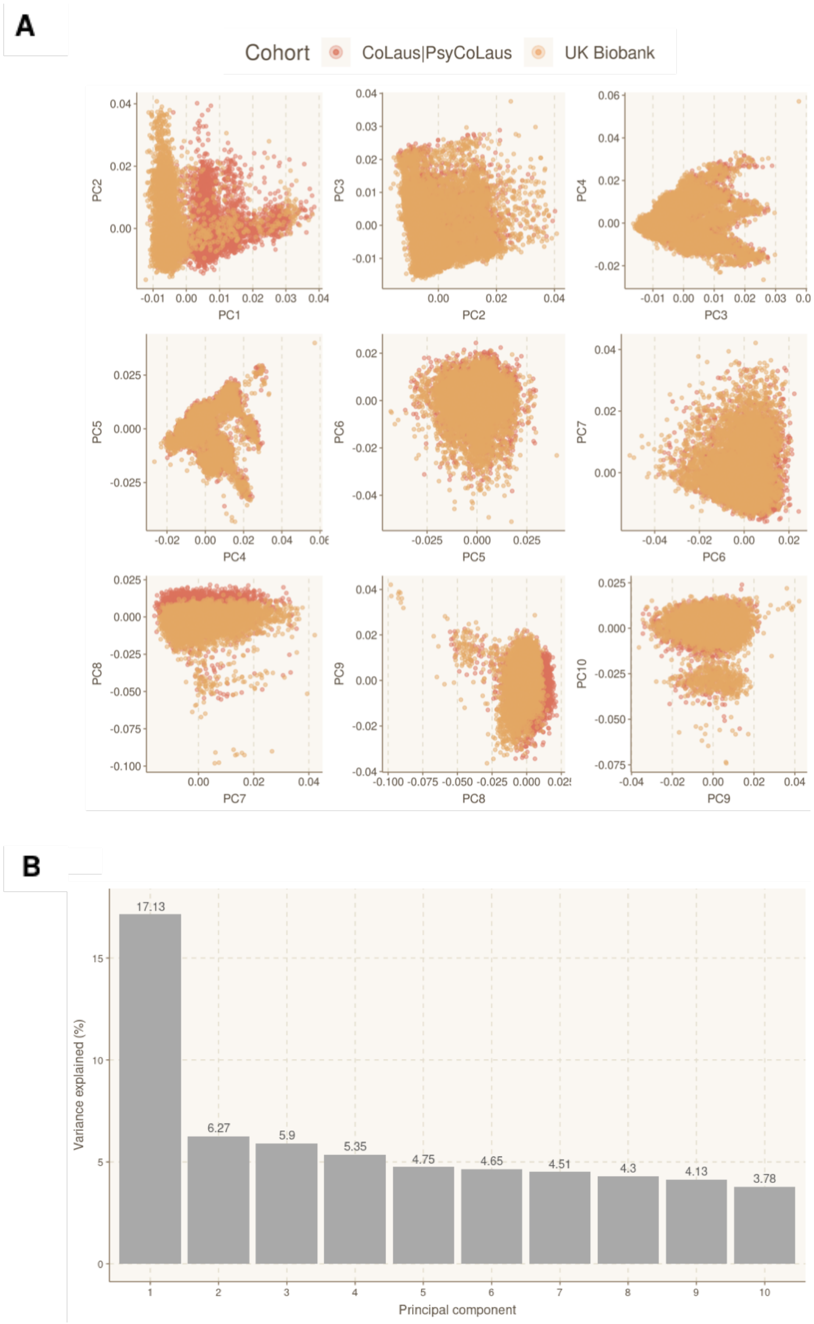

Supplementary Figure 3. Principal component analysis (PCA) of combined genotyping data. A) PCA plot of the first ten PCs of the genotyping data. Samples are colored by cohort. B) Histogram explaining the variance of each PC component. In the histogram, the variance explained by each eigenvalue is labeled on top.
