# Supplementary material for: The combined impact of persistent infections and human genetic variation on C-reactive protein levels
Source: BMC Med. 2022 Nov 1;20:416. doi: 10.1186/s12916-022-02607-7 (PMC9623937; doi:10.1186/s12916-022-02607-7)
Supplement: Supplementary file 4 — Additional file 4: Fig. S4. Distribution of polygenic risk score (PRS) values. Density distribution of standardized PRS values by subcohort (CoLaus|PsyCoLaus and UKB) and across all participants (combined). [file 12916_2022_2607_MOESM4_ESM.pdf]

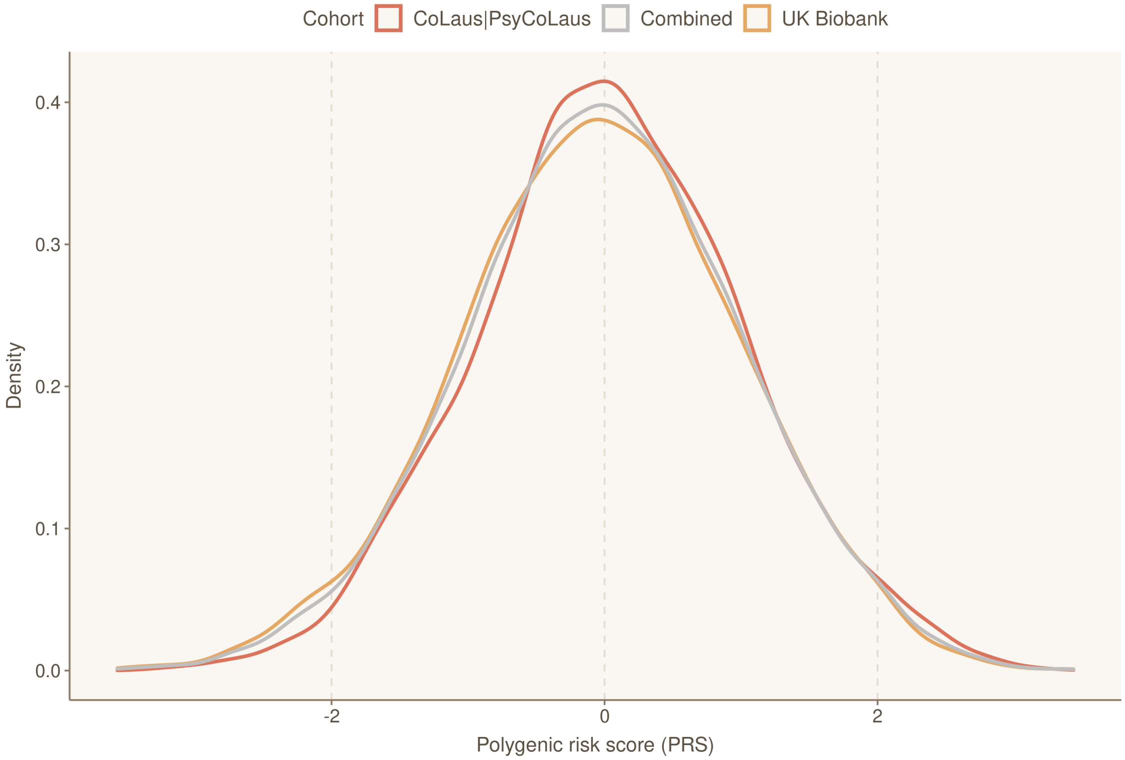


**Supplementary Figure 4. Distribution of polygenic risk score (PRS) values.** Density distribution of standardized PRS values by subcohort (CoLaus|PsyCoLaus and UKB) and across all participants (combined).
