# Supplementary material for: The combined impact of persistent infections and human genetic variation on C-reactive protein levels
Source: BMC Med. 2022 Nov 1;20:416. doi: 10.1186/s12916-022-02607-7 (PMC9623937; doi:10.1186/s12916-022-02607-7)
Supplement: Supplementary file 5 — Additional file 5: Fig. S5. Polygenic risk score for hs-CRP (CRP-PRS) was significantly associated with hs-CRP levels. Scatter plots with linear regression line of polygenic risk scores predicting hs-CRP levels for individuals in the cohort. 95% confidence interval is showed in grey shade. [file 12916_2022_2607_MOESM5_ESM.pdf]

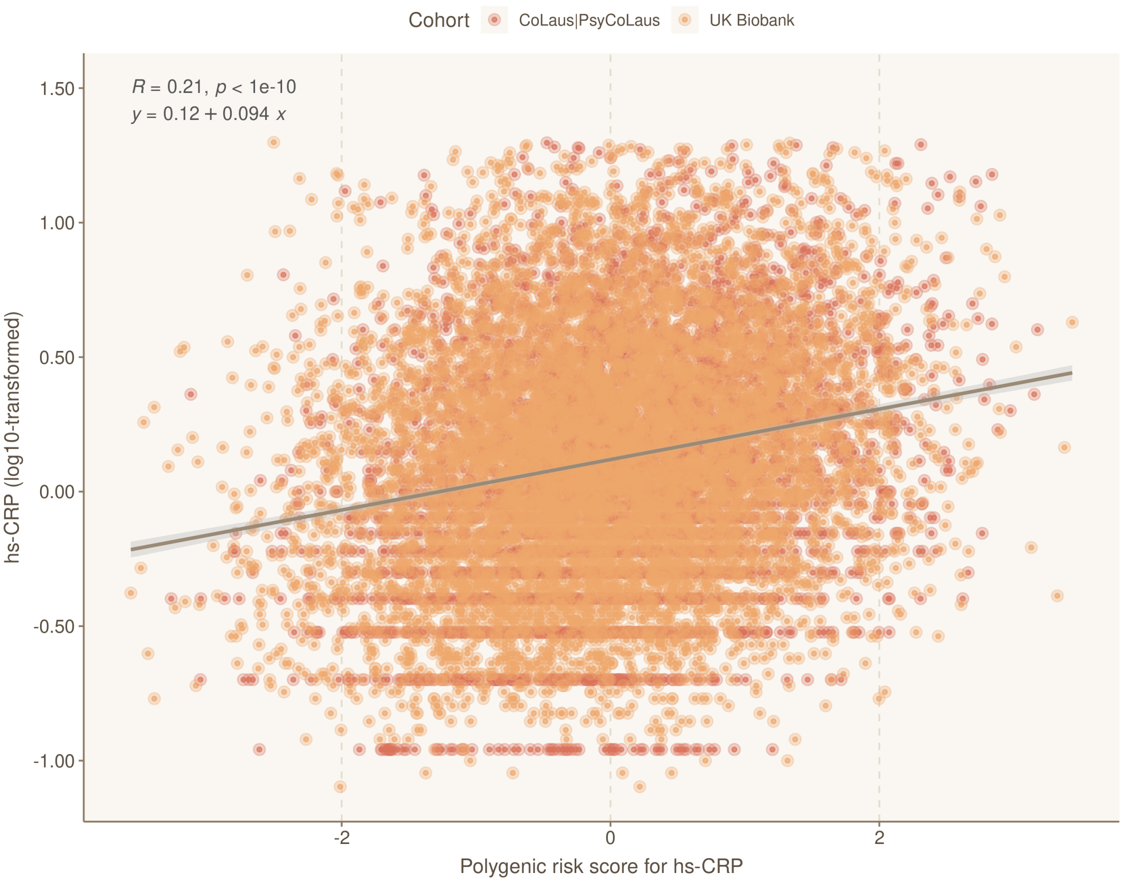

Supplementary Figure 5. Polygenic risk score for hs-CRP (CRP-PRS) was significantly associated with hs-CRP levels. Scatter plots with linear regression line of polygenic risk scores predicting hs-CRP levels for individuals in the cohort. 95% confidence interval is showed in grey shade.
