# Supplementary material for: The combined impact of persistent infections and human genetic variation on C-reactive protein levels
Source: BMC Med. 2022 Nov 1;20:416. doi: 10.1186/s12916-022-02607-7 (PMC9623937; doi:10.1186/s12916-022-02607-7)
Supplement: Supplementary file 6 — Additional file 6: Fig. S6. Seroprevalence of tested antigens in the CoLaus|PsyCoLaus. List of the 27 antigens available from the CoLaus|PsyCoLaus study that are shared with the UK Biobank. The percentages indicate the seroprevalence of antibodies against infectious disease antigens tested using Multiplex Serology platform. The grey boxes indicate the pathogen on which the antigen protein is found, and the family to which the pathogen belongs. [file 12916_2022_2607_MOESM6_ESM.pdf]

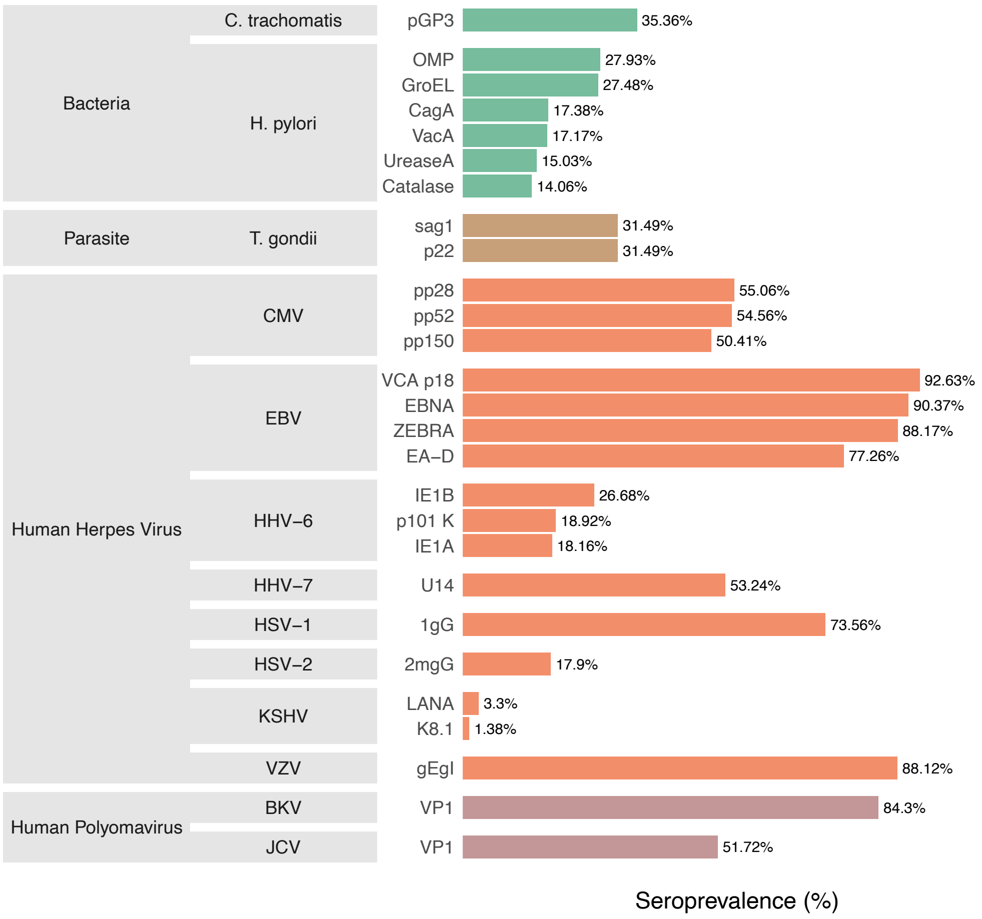

Supplementary Figure 6. Seroprevalence of tested antigens in the CoLaus|PsyCoLaus. List of the 27 antigens available from the CoLaus|PsyCoLaus study that are shared with the UK Biobank. The percentages indicate the seroprevalence of antibodies against infectious disease antigens tested using Multiplex Serology platform. The grey boxes indicate the pathogen on which the antigen protein is found, and the family to which the pathogen belongs.
